# Pollinators on the polar edge of the Ecumene: taxonomy, phylogeography, and ecology of bumble bees from Novaya Zemlya

**DOI:** 10.3897/zookeys.866.35084

**Published:** 2019-07-24

**Authors:** Grigory S. Potapov, Alexander V. Kondakov, Boris Yu. Filippov, Mikhail Yu. Gofarov, Yulia S. Kolosova, Vitaly M. Spitsyn, Alena A. Tomilova, Natalia A. Zubrii, Ivan N. Bolotov

**Affiliations:** 1 Northern Arctic Federal University, 163002, Northern Dvina Emb. 17, Arkhangelsk, Russia Federal Center for Integrated Arctic Research, Russian Academy of Sciences Arkhangelsk Russia; 2 Federal Center for Integrated Arctic Research, Russian Academy of Sciences, 163000, Northern Dvina Emb. 23, Arkhangelsk, Russia Northern Arctic Federal University Arkhangelsk Russia

**Keywords:** Hymenoptera, Apidae, *
Bombus
*, Arctic Ocean archipelagoes, Pleistocene glaciations, mitochondrial DNA

## Abstract

The High Arctic bumble bee fauna is rather poorly known, while a growing body of recent molecular research indicates that several Arctic species may represent endemic lineages with restricted ranges. Such local endemics are in need of special conservation efforts because of the increasing anthropogenic pressure and climate changes. Here, we re-examine the taxonomic and biogeographic affinities of bumble bees from Novaya Zemlya using historical samples and recently collected materials (1895–1925 *vs.* 2015–2017). Three bumble bee species inhabit the Yuzhny (Southern) Island and the southern edge of Severny (Northern) Island of this archipelago: *Bombusglacialis* Friese, 1902, *B.hyperboreus* Schönherr, 1809, and *B.pyrrhopygus* Friese, 1902. *Bombusglacialis* shares three unique COI haplotypes that may indicate its long-term (pre-glacial) persistence on Novaya Zemlya. In contrast, *Bombushyperboreus* and *B.pyrrhopygus* share a rather low molecular divergence from mainland populations, with the same or closely related haplotypes as those from Arctic Siberia and Norway. A brief re-description of *Bombuspyrrhopygus* based on the newly collected topotypes is presented. Habitats, foraging plants and life cycles of bumble bees on Novaya Zemlya are characterized, and possible causes of extremely low bumble bee abundance on the archipelago are discussed. The species-poor bumble bee fauna of Novaya Zemlya is compared with those in other areas throughout the Arctic. The mean bumble bee species richness on the Arctic Ocean islands is three times lower than that in the mainland Arctic areas (3.1 *vs.* 8.6 species per local fauna, respectively). General linear models (GLMs) indicate that this difference can be explained by specific environmental conditions of insular areas. Our findings highlight that the insularity is a significant factor sharply decreasing species richness in bumble bee assemblages on the Arctic Ocean archipelagoes through colder climate (lower summer temperatures), prevalence of harsh Arctic tundra landscapes with poor foraging resources, and in isolation from the mainland.

## Introduction

Novaya Zemlya is an Arctic archipelago comprising two large islands, i.e., the Yuzhny (Southern) and Severny (Northern) islands, and numerous small islets. This huge insular area has a harsh Arctic climate (Coulson et al. 2014). Dwarf-shrub tundra and moss wetlands are the most typical assemblages for the coastal areas of the Yuzhny Island, while rocky mountain tundra covers its central range. Large mountain glaciers occupy the Severny Island, but its southern margin and narrow coastal areas are ice-free and covered by Arctic tundra landscapes (Walker et al. 2005). It was thought that Novaya Zemlya has a low level of endemism of vascular plants and terrestrial animals (Brochmann et al. 2003) and that extensive Pleistocene ice sheets did not cover the Yuzhny Island (Mangerud et al. 2008; Coulson et al. 2014).

The terrestrial invertebrate fauna of the Novaya Zemlya Archipelago is relatively poorly known, because there were few researchers compared with other areas of the Arctic (Coulson et al. 2014). However, several groups of large insects such as bumble bees have attracted the full attention of collectors even during the initial exploration period of Novaya Zemlya (Holmgren 1883; Jacobson 1898). Later, the bumble bee fauna of Novaya Zemlya was examined in a series of taxonomic and ecological works (Friese 1902, 1905, 1908, 1923; Sparre-Schneider 1909; Høeg 1924) and was discussed in subsequent reviews on bumble bees from various northern Palearctic areas (Pittioni 1942, 1943; Løken 1973; Rasmont and Iserbyt 2014; Potapov et al. 2014; Rasmont et al. 2015). Finally, a recent study confirms the status of *Bombusglacialis* as a divergent phylogenetic lineage and a putative endemic species to the Arctic Ocean islands including Novaya Zemlya (Potapov et al. 2018a).

This paper aims to re-examine the taxonomic and biogeographic affinities of bumble bees from Novaya Zemlya using newly collected samples from two sites on the Yuzhny Island. Based on our novel phylogeographic data, we suggest putative historical biogeographic scenarios explaining the origin of bumble bee fauna on Novaya Zemlya and other Arctic Ocean islands. We compare the species richness of bumble bees on the Arctic Ocean islands with that in the mainland Arctic areas and estimate a possible influence of polar climate and harsh landscapes on the low species richness of bumble bee faunas in the High Arctic using general linear modeling approach. Finally, issues concerning the current taxonomy of *Bombusglacialis*, *B.hyperboreus*, *B.pyrrhopygus*, and the entire subgenus Alpinobombus are critically discussed with a special focus to the newly obtained molecular sequence data from Novaya Zemlya and adjacent areas.

## Materials and methods

### Data sampling and morphological study

A bumble bee sample from Novaya Zemlya typically represents a daily sampling effort of a single collector in most cases, while only a few samples represent a bumble bee collection during several days (Table 1). The historical samples of bumble bees from Novaya Zemlya were studied by Grigory S. Potapov in the Natural History Museum [**NHMUK**], London, UK; Tromsø University Museum [**TMU**], Tromsø, Norway; Zoological Museum of Moscow University [**ZMMU**], Moscow, Russia; Zoological Institute of the Russian Academy of Sciences [**ZISP**], Saint Petersburg, Russia. The type locality of *B.hyperboreus* is given according to the database of the Swedish Royal Museum of Natural History (Naturhistoriska riksmuseet) [**NRM**], Stockholm, Sweden.

**Table 1.** Collecting localities and samples of bumble bees from Novaya Zemlya.

Locality	N	E	Date	Collector	Number of specimens	Number of species	Depository
**Recent samples**							
Malye Karmakuly (YI)	72.3992, 52.8671		27.vii.2015	Spitsyn	5	3	RMBH
Malye Karmakuly (YI)	72.3742, 52.7806		28.vii.2015	Spitsyn	4	2	RMBH
Malye Karmakuly (YI)	72.3754, 52.7241		30.vii.2015	Spitsyn	1	1	RMBH
Malye Karmakuly (YI)	72.3739, 52.7167		5.viii.2015	Spitsyn	1	1	RMBH
Malye Karmakuly (YI)	72.4229, 52.8143		6.viii.2015	Spitsyn	1	1	RMBH
Malye Karmakuly (YI)	72.3905, 52.7167		9.viii.2015	Spitsyn	1	1	RMBH
Bezymyannaya Bay (YI)	72.8169, 53.7843		21.vii.2017	Spitsyn	1	1	RMBH
Bezymyannaya Bay (YI)	72.8338, 53.3781		23.vii.2017	Spitsyn	6	2	RMBH
Bezymyannaya Bay (YI)	72.8120, 53.8411		23.vii.2017	Spitsyn	1	1	RMBH
Bezymyannaya Bay (YI)	72.8781, 53.6303		23.vii.2017	Spitsyn	1	1	RMBH
Bezymyannaya Bay (YI)	72.8667, 53.6335		19-21.vii.2017	Spitsyn	2	2	RMBH
Bezymyannaya Bay (YI)	72.8528, 53.7134		19-26.vii.2017	Spitsyn	8	2	RMBH
Bezymyannaya Bay (YI)	72.8335, 53.7339		19-26.vii.2017	Spitsyn	4	3	RMBH
**Mean ± s.e.m.**					**2.77±0.66**	**1.62±0.22**	
**Historical samples**							
n/a	n/a	n/a	n/a	n/a	6	2	NHMUK
Matochkin Shar Strait (YI)*	73.2, 56.4		12.vii.1925	Vakulenko	1	1	HNMUK
n/a	n/a	n/a	n/a	n/a	1	1	TMU
Kostin Shar Strait (YI)*	71.1, 53.7		19.vii.1895	n/a	1	1	TMU
Krestovaya Bay (NI)	74.0, 55.5		10-12.viii.1909	Rusanov	1	1	ZMMU
Matochkin Shar Strait, broadcast station (YI)	73.2, 56.4		3.vii.1924	Tolmachev	1	1	ZMMU
Matochkin Shar Strait, Nochuev Stream (YI)	73.2, 56.3		31.vii.1925	Vakulenko	1	1	ZMMU
Kostin Shar Strait, Propashchaya Bay (YI)*	71.1, 53.7		16.viii.1925	Pokrovskiy	1	1	ZMMU
Matochkin Shar Strait (YI)*	73.2, 56.4		11.viii.1925	Pokrovskiy	1	1	ZMMU
Malye Karmakuly (YI)	72.3, 52.7		23.vii.1896	Jacobson	10	2	ZISP
Verkhnyaya Tyulenya Bay (NI)*	73.3, 56.0		9.vii.1901	Timofeev	9	1	ZISP
Chekin Bay (NI)	73.5, 57.0		27.vii.1901	Timofeev	2	2	ZISP
Novosiltsev Lake (NI)*	73.6, 56.3		2.viii.1901	Timofeev	1	1	ZISP
Peschanka River (YI)	73.2, 53.6		22.viii.1902	n/a	1	1	ZISP
Bychkov River (NI)*	73.5, 55.0		5.viii.1907	n/a	1	1	ZISP
Krestovaya Bay (NI)	74.0, 55.5		10-12.viii.1909	Rusanov	5	1	ZISP
Krestovaya Bay (NI)	74.0, 55.5		22.vii.1910	Sosnovskiy	7	1	ZISP
Kostin Shar Strait, Propashchaya Bay (YI)*	71.1, 53.7		1-9.viii.1913	Skribov	2	2	ZISP
Matochkin Shar Strait, broadcast station (YI)	73.2, 56.4		21.vi.-11.viii.1924	Tolmachev	6	2	ZISP
Matochkin Shar Strait (YI)*	73.2, 56.4		13-15.vii.1924	Tolmachev	4	3	ZISP
Matochkin Shar Strait, Nochuev Stream (YI)	73.2, 56.3		23.vii.1924	Tolmachev	2	2	ZISP
Matochkin Shar Strait, Poperechniy Cape (YI)	73.2, 56.1		5.viii.1924	Tolmachev	5	2	ZISP
Matochkin Shar Strait (YI)*	73.2, 56.4		2.vii.1925	Tolmachev	14	1	ZISP
Matochkin Shar Strait, Nochuev Stream (YI)	73.2, 56.3		18.vii.1925	Tolmachev	2	1	ZISP
Matochkin Shar Strait, Nochuev Stream (YI)	73.2, 56.3		1.viii.1925	Tolmachev	1	1	ZISP
Plateau (YI)*	73.2, 56.3		1.viii.1925	Tolmachev	1	1	ZISP
Matochkin Shar Strait, coast (YI)*	73.2, 56.4		9.vi.1925	Vakulenko	1	1	ZISP
Matochkin Shar Strait, Blizhnyaya Mountain (YI)*	73.2, 56.5		21.vi.1925	Vakulenko	3	1	ZISP
Matochkin Shar Strait, observatory (YI)	73.2, 56.4		29.vi.1925	Vakulenko	1	1	ZISP
Matochkin Shar Strait (YI)*	73.2, 56.4		6.-15.vii.1925	Vakulenko	7	3	ZISP
Belushya Bay (NI)	73.3, 56.0		5.-7.vii.1925	Vakulenko	2	1	ZISP
**Mean ± s.e.m.**					**3.26±0.59**	**1.35±0.11**	

Key: YI – Yuzhny Island, NI – Severny Island of the Novaya Zemlya Archipelago, *Coordinates of these localities are approximate, n/a – not available (locality, date, or collector are unknown).

**Figure 1.**
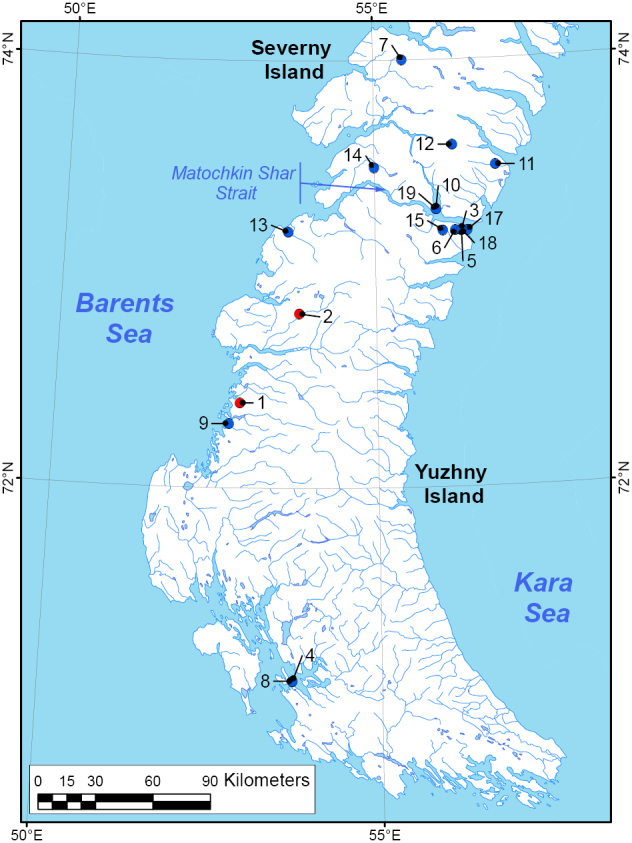
Map of bumble bee collecting localities on Novaya Zemlya (YI – Yuzhny Island, NI – Severny Island). Recent samples (red circles): 1 – Malye Karmakuly (YI); 2 – Bezymyannaya Bay (YI). Historical samples (blue circles): 3 – Matochkin Shar Strait (YI); 4 – Kostin Shar Strait (YI); 5 – Matochkin Shar Strait, broadcast station (YI); 6 – Matochkin Shar Strait, Nochuev Stream (YI); 7 – Krestovaya Bay (NI); 8 - Kostin Shar Strait, Propashchaya Bay (YI); 9 – Malye Karmakuly (YI); 10 – Verkhnyaya Tyulenya Bay (NI); 11 – Chekin Bay (NI); 12 – Novosiltsev Lake (NI); 13 – Peschanka River (YI); 14 – Bychkov River (NI), 15 – Matochkin Shar Strait, Poperechniy Cape (YI); 16 – Matochkin Shar Strait, coast (YI); 17 – Matochkin Shar Strait, Blizhnyaya Mountain (YI); 18 – Matochkin Shar Strait, observatory (YI).

**Figure 2.**
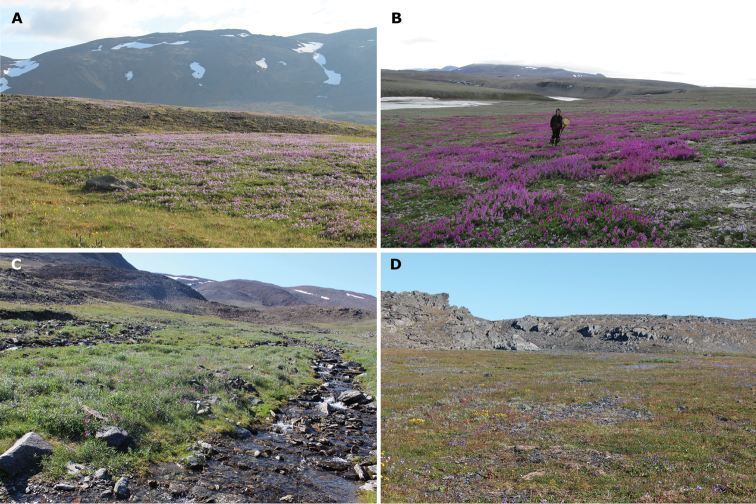
Habitats of bumble bees on Novaya Zemlya (Yuzhny Island). (**A**) Herb tundra patch with Alpine milkvetch (*Astragalusalpinus*), Bezymyannaya Bay, 20.vii.2017. (**B**) Herb tundra patch with Arctic sweetvetch (*Hedysarumarcticum*), Bezymyannaya Bay, 29.vii.2017. (**C**) Meadow-like association with dwarf fireweed (*Chamaenerionlatifolium*) along a stream valley, Bezymyannaya Bay, 26.vii.2017. (**D**) Meadow-like association on a mountain terrace, Malye Karmakuly, 28.vii.2015. Photographs by Vitaly M. Spitsyn (**A, C–D**), Elena Y. Churakova (**B**).

**Figure 3.**
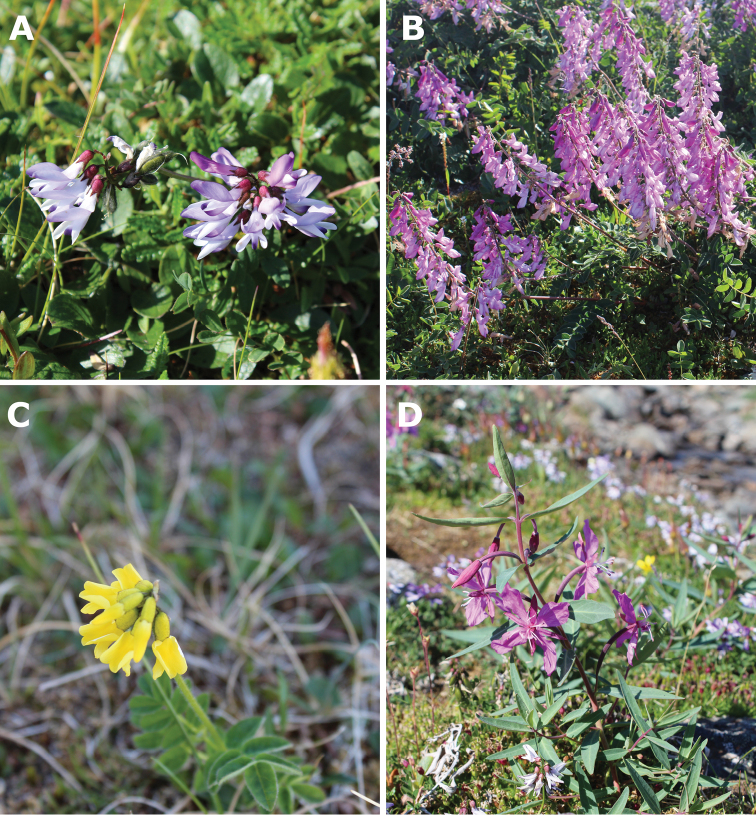
Primary foraging resources of bumble bees on Novaya Zemlya (Yuzhny Island, Bezymyannaya Bay). (**A**) Alpine milkvetch (*Astragalusalpinus*), 26.vii.2017. (**B**) Arctic sweetvetch (*Hedysarumarcticum*), 27.vii.2017. (**C**) Tundra milkvetch (*Astragalusumbellatus*), 20.vii.2017. (**D**) Dwarf fireweed (*Chamaenerionlatifolium*), 26.vii.2017. Photographs by Vitaly M. Spitsyn.

The recent samples of bumble bees were collected by Vitaly M. Spitsyn from two sites on the Yuzhny Island of Novaya Zemlya: Malye Karmakuly Station, 27.vii-9.viii.2015 (*N* = 13 specimens); and Bezymyannaya Bay, 19–26.vii.2017 (*N* = 23 specimens) (Figs 1–3, Tables 1–2, and Suppl. material 2: Table S3). These samples were pinned and deposited in the Russian Museum of the Biodiversity Hotspots [**RMBH**] of the Federal Center for Integrated Arctic Research of the Russian Academy of Sciences (Arkhangelsk, Russia).

**Table 2.** Bumble bee assemblages (total number of specimens) in historical and recent collections from Novaya Zemlya.

**Locality**	**Year**	*** Bombus glacialis ***		*** Bombus pyrrhopygus ***		*** Bombus hyperboreus ***	
		***N***	**Caste composite**	***N***	**Caste composite**	***N***	**Caste composite**
**Recent samples**							
Malye Karmakuly (YI)	2015	7	4♀, 1♂, 2☿	5	4♀, 1☿	1	1♀
Bezymyannaya Bay (YI)	2017	16	1♀, 15☿	5	4♀, 1☿	2	2♀
**Total**		**23**	**5**♀, **1**♂, **17**☿	**10**	**8**♀, **2**☿	**3**	**3**♀
**Historical samples**							
Kostin Shar Strait (YI)	1895	1	1♀	–	–	–	–
Malye Karmakuly (YI)	1896	–	–	8	7♂,1☿	2	2♀
Verkhnyaya Tyulenya Bay (NI)	1901	9	9☿	–	–	–	–
Chekin Bay (NI)	1901	1	1♀	–	–	1	1♀
Novosiltsev Lake (NI)	1901	–	–	–	–	1	1♀
Peschanka River (YI)	1902	1	1♂	–	–	–	–
Bychkov River (NI)	1907	–	–	1	1♂	–	–
Krestovaya Bay (NI)	1909	5	1♀, 4♂,	–	–	1	1♂
Krestovaya Bay (NI)	1910	7	1♀, 4♂, 2☿,	–	–	–	
Kostin Shar Strait, Propashchaya Bay (YI)	1913	–	–	1	1♂	1	1♀
Matochkin Shar Strait (YI)	1924	2	2♀	1	1♀	1	1♀
Matochkin Shar Strait, broadcast station (YI)	1924	6	6♀	1	1♀		
Matochkin Shar Strait, Nochuev Stream (YI)	1924	1	1♀	–	–	1	1♀
Matochkin Shar Strait, Poperechniy Cape (YI)	1924	4	1♀, 2♂, 1☿	1	1☿	–	–
Matochkin Shar Strait	1925	21	11♀, 10☿	1	1♀	2	2♀
Matochkin Shar Strait, Nochuev Stream (YI)	1925	4	1♀, 1♂, 2☿	–	–	–	–
Matochkin Shar Strait, Blizhnyaya Mountain (YI)	1925	3	3♀	–	–	–	–
Matochkin Shar Strait, observatory (YI)	1925	–	–	–	–	1	1♀
Kostin Shar Strait, Propastshaya Bay (YI)	1925	–	–	–	–	1	1♀
Belushya Bay (NI)	1925	2	2♀	–	–	–	–
**Total**		**67**	**31**♀, **12**♂, **24**☿	**14**	**3**♀, **9**♂, **2**☿	**12**	**11**♀, **1**♂

Key: YI – Yuzhny Island, NI – Severny Island of the Novaya Zemlya Archipelago. “–” indicates the lack of a species in a given sample.

The bumble bee specimens were studied using a stereomicroscope Solo 2070 (Carton Optical (Siam) Co., Ltd., Thailand). For the morphological study of samples, we applied a standard approach and terminology described by Løken (1973) and Williams et al. (2008, 2014). Images of the morphological details were taken using a stereomicroscope Leica EZ4D (Leica Microsystems GmbH, Germany).

### Laboratory protocols and searching for the nearest neighbor sequences

We obtained new sequences of the *cytochrome c oxidase subunit I* (COI) gene from 27 bumble bee specimens, including the topotypes of *Bombuspyrrhopygus* (Table 3). The laboratory protocols were as described in Potapov et al. (2018a). Resulting COI gene sequences were checked manually using a sequence alignment editor (BioEdit v. 7.2.5; Hall 1999). Phylogenetic relations of the COI haplotypes were checked with the BOLD COI Full Database (BOLD thereafter) (Ratnasingham and Hebert 2007) and with the NCBI’s GenBank using a Basic Local Alignment Search Tool, BLAST (Altschul et al. 1990).

**Table 3.** List of COI sequences for bumble bee specimens from Novaya Zemlya (Yuzhny Island). The list of additional sequences of bumble bees from other regions used in this study is presented in Suppl. material 1, Table S1.

Species	COI haplotype code	GenBank accession number	Specimen voucher [RMBH]	Locality
* B. glacialis *	GL1	KY202838	BMB78	Malye Karmakuly
* B. glacialis *	GL1	KY202839	BMB79	Malye Karmakuly
* B. glacialis *	GL1	KY202840	BMB80	Malye Karmakuly
* B. glacialis *	GL1	KY202841	BMB82	Malye Karmakuly
* B. glacialis *	GL1	KY202842	BMB83	Malye Karmakuly
* B. glacialis *	GL1	KY202843	BMB84	Malye Karmakuly
* B. glacialis *	GL1	MK530672	BMB158	Bezymyannaya Bay
* B. glacialis *	GL1	MK530674	BMB162	Bezymyannaya Bay
* B. glacialis *	GL1	MK530669	BMB153	Bezymyannaya Bay
* B. glacialis *	GL1	MK530670	BMB154	Bezymyannaya Bay
* B. glacialis *	GL1	MK530675	BMB164	Bezymyannaya Bay
* B. glacialis *	GL1	MK530676	BMB165	Bezymyannaya Bay
* B. glacialis *	GL1	MK530677	BMB166	Bezymyannaya Bay
* B. glacialis *	GL1	MK530678	BMB167	Bezymyannaya Bay
* B. glacialis *	GL2	MK530671	BMB157	Bezymyannaya Bay
* B. glacialis *	GL2	MK530673	BMB161	Bezymyannaya Bay
* B. glacialis *	GL3	MK530683	BMB172	Bezymyannaya Bay
*B.pyrrhopygus* [Topotype]	PY1	MK530667	BMB88	Malye Karmakuly
*B.pyrrhopygus* [Topotype]	PY1	MK530668	BMB90	Malye Karmakuly
* B. pyrrhopygus *	PY1	MK530679	BMB168	Bezymyannaya Bay
* B. pyrrhopygus *	PY1	MK530680	BMB169	Bezymyannaya Bay
* B. pyrrhopygus *	PY1	MK530681	BMB170	Bezymyannaya Bay
* B. pyrrhopygus *	PY1	MK530682	BMB171	Bezymyannaya Bay
* B. pyrrhopygus *	PY1	MK530684	BMB173	Bezymyannaya Bay
* B. hyperboreus *	HY1	MK530666	BMB87	Malye Karmakuly
* B. hyperboreus *	HY2	MK530685	BMB174	Bezymyannaya Bay
* B. hyperboreus *	HY2	MK530686	BMB175	Bezymyannaya Bay

### Phylogeographic analyses

We used a median-joining network approach using Network v. 4.6.1.3 with default settings (Bandelt et al. 1999). Additional COI sequences of *Bombuspyrrhopygus*, *B.hyperboreus* and *B.natvigi* were obtained from the BOLD and GenBank databases (*N* = 26; Suppl. material 1, Table S1). The alignment of COI sequences was performed using the ClustalW algorithm implemented in MEGA7 (Kumar et al. 2016).

### Phylogenetic analyses

For phylogenetic analyses, we used the dataset with unique COI haplotypes of *Alpinobombus* taxa from Novaya Zemlya (Table 3) and other areas (*N* = 43; Suppl. material 1, Table S2). *Bombusignitus*, *B.terrestrisaudax*, and *B.cryptarum* were used as outgroup (GenBank acc. nos. HQ228365, KT074036, and AY530013, respectively). The COI sequences were aligned using the MUSCLE algorithm of MEGA7 (Kumar et al. 2016). The phylogenetic modeling was performed with IQ-TREE (Nguyen et al. 2015) through an online web server (http://iqtree.cibiv.univie.ac.at) (Trifinopoulos et al. 2016). The best-fit evolutionary model (K3Pu+F+G4) was identified with Model Finder based on Bayesian Information Criterion (BIC) (Kalyaanamoorthy et al. 2017). Bootstrap support (BS) values were estimated by means of an ultrafast bootstrap (UFBoot2) approach (Hoang et al. 2018). We used IQ-TREE software, because it achieves the best likelihoods compared with other similar phylogenetic programs (Zhou et al. 2018).

### Species delimitation modeling

Molecular Operational Taxonomic Units (MOTUs) for the subgenus Alpinobombus were obtained using the multi-rate Poisson tree processes (mPTP) model of Kapli et al. (2017) for single-locus species delimitation through online mPTP server (http://mptp.h-its.org). A phylogenetic input tree was obtained from IQ-TREE analysis (see above). The mean genetic divergences (uncorrected *p*-distances) between COI haplotypes were computed in MEGA7 (Kumar et al. 2016).

### Species richness modeling

To estimate the possible role of climatic parameters and insular environment for the bumble bee species richness throughout the Arctic, we applied the general linear models (GLMs; Statistica v. 13.3, Stat Soft Inc., USA). We used species richness plotted against mean air temperature as a covariate and geographic position as a factor with two levels (island *vs.* mainland) (Bolotov et al. 2018). Additionally, we computed models using type of biome as a factor with three levels (Arctic tundra *vs.* tundra *vs.* forest tundra). Monthly and annual mean air temperatures were obtained from the CRU TS v. 4.01 climate database (Climatic Research Unit, University of East Anglia) as gridded variables (0.5° resolution), which were based on weather station records during the period from 1 January 1901 to 31 December 2010 (Harris et al. 2014). Estimations of bumble bee species richness in various sites throughout the Arctic Ocean islands and the mainland were compiled from the body of reliable literature sources. The GLMs were simplified to the minimal adequate models using sequential exclusion of insignificant factors from the model (Crawley 2002). Correlation of species richness with climatic and geographic variables was calculated using Spearman’s coefficients with Statistica v. 13.3.

## Results

### Bumble bee assemblages on Novaya Zemlya

Bumble bees are not abundant on Novaya Zemlya, with the mean value of 2.77 and 3.26 specimens per recent and historical sample, respectively (no significant differences, Mann-Whitney test: *U* = 189, *N*_recent_ = 13, *N*_historical_ = 31, *P* = 0.74) (Table 1). While three bumble bee species are known from Novaya Zemlya, i.e., *Bombusglacialis*, *B.pyrrhopygus* and *B.hyperboreus* (Table 2), the mean number of recorded species per sample is 1.62 and 1.35 in recent and historical samples, respectively (no significant differences, Mann-Whitney test: *U* = 164, *N*_recent_ = 13, *N*_historical_ = 31, *P* = 0.25) (Table 1). Based on the recent and historical samples, *Bombusglacialis* seems to be the most commonly occurring species, while *B.pyrrhopygus* and *B.hyperboreus* have lower abundance (Table 2).

### Bumble bee habitats and primary foraging resources on Novaya Zemlya

The recent samples of bumble bees were collected in three habitat types, representing rather small patches within a continuous mountain tundra landscape: (1) meadow-like associations (17 specimens, 47.2% of a total sample), (2) herb tundra patches with *Astragalusalpinus* (16 specimens, 44.4% of a total sample), and (3) herb tundra patches with *Hedysarumarcticum* (3 specimens, 8.3% of a total sample) (Fig. 2 and Suppl. material 2, Table S3). Bumble bees were not recorded beyond these types of habitats (Vitaly M. Spitsyn, personal observations, 2015–2017). The bumble bees in recent samples were primarily collected from three legume species (*Astragalusalpinus*, *A.umbellatus*, and *Hedysarumarcticum*), and one willowherb species (*Chamaenerionlatifolium*) (Fig. 3). These four plant species seems to be the most important foraging resources for bumble bees in Malye Karmakuly and Bezymyannaya Bay.

### Phylogeny and species delimitation model for the subgenus Alpinobombus

**Figure 4.**
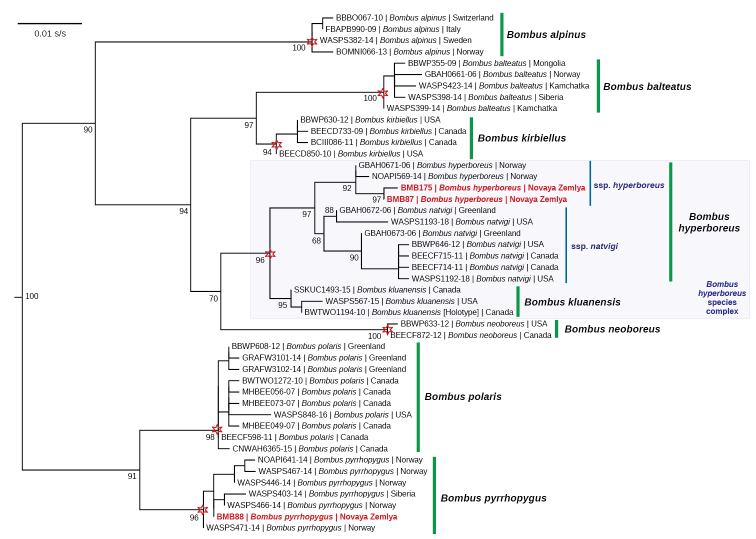
Maximum likelihood (IQ-TREE) phylogeny of the subgenus Alpinobombus based on the COI gene haplotypes. The red asterisks indicate the putative species-level clades supported by mPTP species-delimitation model. The black numbers near nodes are ultrafast bootstrap support values. The haplotypes from Novaya Zemlya are colored red. The *Bombushyperboreus* species complex with two valid species is colored light blue. Outgroup is not shown.

The maximum likelihood phylogeny reveals that two COI haplotypes of *Bombushyperboreus* from Novaya Zemlya cluster together with those from Norway (Fig. 4). The mPTP species-delimitation model supports almost all valid species in this genus, but the clade containing haplotypes of *Bombushyperboreus*, *B.natvigi*, and *B.kluanensis* was considered a single MOTU (Fig. 4). The mean uncorrected COI *p*-distance between *Bombushyperboreus* and *B.natvigi* is 1.6% (rather intraspecific difference), while those between these taxa and *B.kluanensis* are 2.1–2.4% (rather interspecific differences).

### Phylogeography

**Figure 5.**
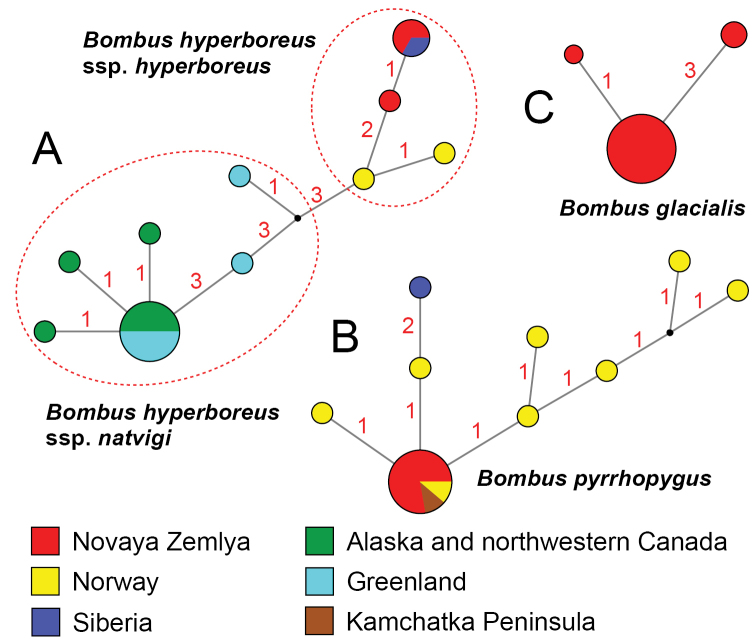
Median-joining haplotype networks of the available COI sequences of bumble bees from Novaya Zemlya and other Arctic areas. (**A)***Bombushyperboreus*. (**B)***B.pyrrhopygus*. (**C)***B.glacialis*. The circle size is proportional to the number of available sequences belonging to a certain haplotype (smallest circle = one sequence). The small black dots indicate hypothetical ancestral haplotypes. Red numbers near branches indicate the number of nucleotide substitutions between haplotypes.

**Figure 6.**
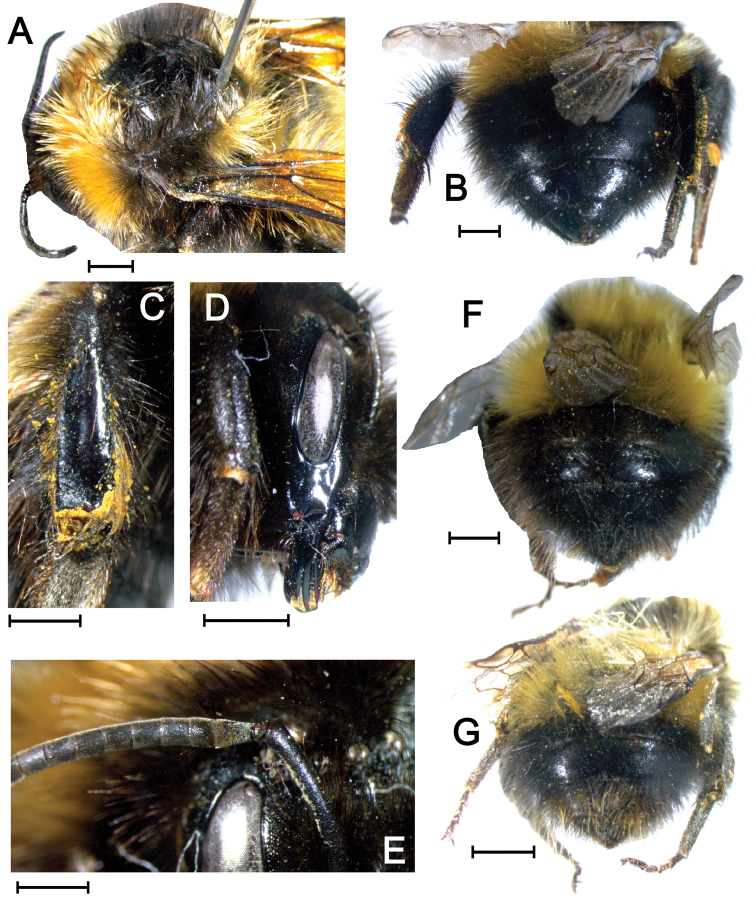
Morphological patterns of *Bombuspyrrhopygus* from Malye Karmakuly, Yuzhny Island, Novaya Zemlya: (**A)** Thorax (prospective topotype RMBH BMB90, queen). (**B)** Metasoma (same topotype queen). (**C)** Hind tibia (same topotype queen). (**D)** Surface of malar space (same topotype queen). (**E)** Flagellum (same topotype queen). (**F)** Metasoma (RMBH BMB88, worker). (**G)** Metasoma (RMBH BMB86, worker). Scale bars 2 mm (A-D, F-G); 1 mm (E). Photographs by Grigory S. Potapov.

**Figure 7.**
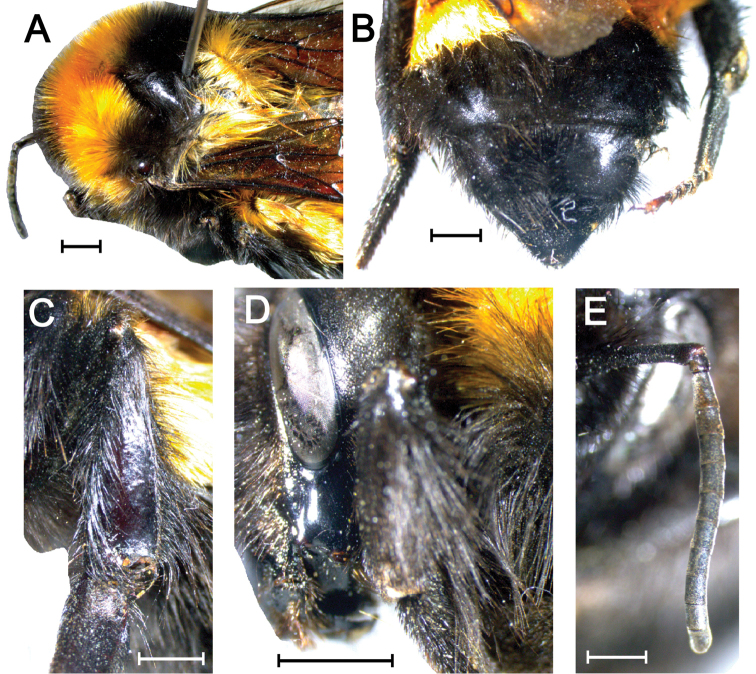
Morphological patterns of *Bombushyperboreus* from Malye Karmakuly, Yuzhny Island, Novaya Zemlya (RMBH BMB87, queen). (**A)** Thorax. (**B)** Metasoma. (**C)** Hind tibia. (**D)** Surface of malar space. (**E)** Flagellum. Scale bars 2 mm (**A-D**); 1 mm (**E**). Photographs Grigory S. Potapov.

*Bombushyperboreus* and *B.pyrrhopygus* from Novaya Zemlya share a low molecular divergence from mainland populations, with the same or closely related haplotypes as those from Arctic Siberia and Norway (Fig. 5A–B). In particular, *Bombuspyrrhopygus* from Novaya Zemlya (Fig. 6) shares a single COI haplotype, which also occurs in Norway and Kamchatka (Fig. 5B). *Bombushyperboreus* from Novaya Zemlya (Fig. 7) shares two COI haplotypes, one of which is also known from the Arctic Siberia (Yakutia), while the second haplotype was not recorded anywhere, but is genetically close to the Norwegian lineage (Fig. 5A). *Bombusglacialis* shares three unique COI haplotypes (Fig. 5C). The first haplotype (GL1) was found in 14 specimens from both recent localities, while the other two haplotypes were recorded only in three specimens from Bezymyannaya Bay (Table 3).

### Bumble bee species richness in the Arctic

The number of bumble bee species on islands of the Arctic Ocean varies from one (Devon Island, Canadian Arctic Archipelago) to seven (Iceland) species, while local faunas in the mainland Arctic areas contains from three (Taymyr Peninsula, Arctic Siberia) to 15 (Pechora River Delta in Arctic European Russia) species (Table 4). We found that the mean bumble bee species richness on the Arctic islands is three times lower than that in the mainland Arctic areas: 3.1 *vs.* 8.6 species per local fauna, respectively (Mann-Whitney test: *U* = 16.5, *N*_island_ = 14, *N*_mailand_ = 16, *P* = 0.0001) (Table 4). The mean temperature of July in the Arctic Ocean island localities is almost two times lower than that in the mainland Arctic localities: 6.7 °C *vs.* 12.0 °C, respectively (Mann-Whitney test: *U* = 22.0, *N*_island_ = 14, *N*_mailand_ = 16, *P* = 0.0002) (Table 4). The annual mean temperature in the insular localities is also slightly lower than that in the mainland localities: -11.7 °C *vs.* -7.5 °C, respectively (Mann-Whitney test: *U* = 64.0, *N*_island_ = 14, *N*_mailand_ = 16, *P* = 0. 0472) (Table 4).

**Table 4.** Species richness of bumble bees on the Arctic Ocean islands and the mainland.

Region	Latitude	Longitude	Biome type**	JMT, °C*	AMT, °C*	Number of species	References
**Islands**							
Novaya Zemlya	72.3N, 52.8E		Arctic tundra	10.42	-7.48	3	This study
Vaigach Island	70.2N, 59.0E		Tundra	11.38	-7.00	5	Potapov et al. (2017)
Kolguev Island	68.8N, 49.2E		Tundra	13.45	-3.42	5	Kolosova and Potapov (2011); Potapov et al. (2014)
Wrangel Island	71.0N, 178.5W		Arctic tundra	2.29	-12.18	3	Berezin (1990); Proshchalykin and Kupianskaya (2005)
Banks Island	71.5N, 123.8W		Arctic tundra	4.45	-14.21	2	Williams et al. (2014)
Victoria Island	69.1N, 105.0W		Tundra	7.46	-14.99	4	Williams et al. (2014)
Prince Patrick Island	76.1N, 121.7W		Arctic tundra	3.52	-17.54	3	Williams et al. (2014)
Melville Island	75.2N, 109.0W		Arctic tundra	4.03	-17.33	1	Williams et al. (2014)
Devon Island	74.6N, 82.4W		Arctic tundra	3.29	-17.69	1	Chernov (2004)
Baffin Island	72.6N, 77.9W		Arctic tundra	4.41	-15.98	5	Williams et al. (2014)
Southampton Island	64.2N, 83.2W		Arctic tundra	8.55	-11.66	4	Williams et al. (2014)
Ellesmere Island	80.0N, 85.9W		Arctic tundra	4.41	-20.38	4	Williams et al. (2014)
Greenland	69.2N, 50.0W		Arctic tundra	5.44	-8.05	2	Pape (1983); Vilhelmsen (2015)
Iceland	64.0N, 21.6W		Tundra	10.45	3.69	1[+6]***	Prŷs-Jones et al. (2016); Potapov et al. (2018b)
**Mean ± s.e.m.**				**6.7±1.0**	-**11.7±1.8**	**3.1±0.4**	
**Mainland**							
Finnmark, Norway	70.8N, 29.0E		Tundra	11.66	-0.85	8	Løken (1973, 1984)
Kola Peninsula (north)	69.0N, 33.1E		Tundra	12.34	-0.19	7	Paukkunen and Kozlov (2015)
Kanin Peninsula (north)	67.8N, 44.1E		Tundra	14.25	-1.57	5	Kolosova and Potapov (2011); Potapov et al. (2014)
Kanin Peninsula (south)	66.6N, 44.6E		Forest tundra	14.65	-1.27	14	Kolosova and Potapov (2011); Potapov et al. (2014)
Pechora River Delta	67.6N, 53.0E		Forest tundra	13.09	-3.72	15	Ross (2000); Kolosova and Potapov (2011); Potapov et al. (2014)
Pymvashor Hot Springs	67.0N, 60.5E		Tundra	12.82	-5.55	12	Kolosova et al. (2016)
Yugorsky Peninsula	69.7N, 61.6E		Tundra	11.60	-7.08	11	Potapov et al. (2017)
Polar Ural	66.9N, 65.7E		Tundra	12.71	-6.48	5	Kaygorodova (1978); Bogacheva and Shalaumova (1990); Olshvang (1992)
Taymyr Peninsula (south)	73.2N, 90.5E		Tundra	10.49	-12.49	3	Chernov (1978)
Tiksi, Yakutia	71.6N, 128.8E		Tundra	13.88	-16.54	6	Davydova (2003)
Indigirka River Delta	71.0N, 149.0E		Tundra	10.67	-14.45	8	Shelokhovskaya (2009)
Chukotka Peninsula	64.7N, 177.4E		Tundra	10.70	-7.54	7	Proshchalykin and Kupianskaya (2005)
Alaska (north)	69.4N, 152.1W		Tundra	10.66	-9.17	13	Williams et al. (2014)
Mackenzie River Delta	67.5N, 134.1W		Tundra	13.92	-8.98	14	Williams et al. (2014)
Coppermine River Delta	67.7N, 115.1W		Tundra	9.75	-11.45	4	Williams et al. (2014)
Bathurst Inlet	66.5N, 108.0W		Tundra	9.59	-12.87	5	Williams et al. (2014)
**Mean ± s.e.m.**				**12.0±0.4**	-**7.5±1.3**	**8.6±1.0**	

Key: *JMT – July mean temperature; AMT – annual mean temperature. Mean temperature values (1901-2010) were obtained from the CRU TS v. 4.01 climate database (Climatic Research Unit, University of East Anglia). **Types of biomes were determined using available classification schemes (Aleksandrova 1976; Olson et al. 2001; Walker et al. 2005). ***The one native bumble bee species, *Bombusjonellus*, inhabits Iceland; the other six species have recently colonized this island via human-mediated dispersal or direct introduction events (Prŷs-Jones et al. 2016; Potapov et al. 2018b). We used only the one native species in our subsequent calculations and species richness modeling.

The bumble bee species richness is correlated with latitude (Spearman *R* = -0.39, *N* = 30, *P* = 0.0325), annual mean air temperature (Spearman *R* = 0.4219, *N* = 30, *P* = 0.0202), and July mean air temperature (Spearman *R* = 0.7537, *N* = 30, *P* < 0.0001). As the mean temperature of July was found to be the most influential factor based on the nonparametric correlation analyses, we have used this parameter in the general linear models (GLMs) (Table 5). Results of the GLMs indicate that the bumble bee species richness in the Arctic is significantly influenced by the mean temperature of July (Table 5). The island position is an indirect significant factor, which is associated with the lower mean temperature of July in the insular areas. Furthermore, the species richness of bumble bees is influenced by type of biome independently of the mean temperature of July.

**Table 5.** Results of general linear models (GLMs) of bumble bee species richness on the Arctic Ocean islands and the mainland. Regression models were simplified to the minimal adequate models (Crawley 2002).

**Response variable**	**Source**	***SS***	***d.f.***	***F***	***P***
Species richness (R^2^ = 0.72)	Intercept	–	–	–	n.s.
	July mean temperature	734.75	1	87.02	<0.0001
	Geographic position (island vs mainland)	–	–	–	n.s.
	July mean temperature × Geographic position	60.11	1	7.12	0.0125
	Error	236.42	28		
Species richness (R^2^ = 0.72)	Intercept	888.43	1	102.25	<0.0001
	July mean temperature	–	–	–	n.s.
	Type of biome	259.40	2	14.93	<0.0001
	July mean temperature × Type of biome	–	–	–	n.s.
	Error	234.60	27		

## Taxonomic account

### Order Hymenoptera

#### Family Apidae

##### Genus *Bombus* Latreille, 1802

###### Subgenus Alpinobombus

Taxon classificationAnimaliaHymenopteraApidae

Skorikov, 1914

07931864-b0e3-545e-b018-2cfcc0083b31

####### Type species.

*Apisalpina* Linnaeus (by subsequent designation)

###### Bombus pyrrhopygus

Taxon classificationAnimaliaHymenopteraApidae

Friese, 1902

b7bcb67a-4109-5a8c-966d-e5170ef2129e

Fig. 6A–G

Bombus kirbyellus subsp. pyrrhopygus Friese (1902): 495; Friese (1905): 515.
Bombus kirbyellus var. pleuralis sensu Friese, 1902 non Nylander, 1848: Friese (1902): 495; Friese (1905): 515; Friese (1923): 4.
Bombus kirbyellus var. cinctus Friese (1911a): 456; Friese (1923): 6.
Bombus kirbyellus var. cinctellus Friese (1911a): 456; Friese (1923): 6.
Bombus alpinus var. diabolicus Friese (1911b): 571.
Bombus alpinus var. pretiosus Friese (1911b): 571.
Bombus kirbyellus var. semljaensis Friese (1923): 4.
Bombus arcticus var. alpiniformis Richards (1931): 13.

####### Type locality.

Nowaja Semlja [Novaya Zemlya] (Friese 1902). It is most likely that the exact type locality was situated somewhere around the Malye Karmakuly Station, because the type series has been collected by G.G. Jacobson in the year 1896 (Friese 1905). Jacobson (1898) noted that he collected the sample of bumble bees near Malye Karmakuly.

####### Type.

Whereabouts unknown. Rasmussen and Ascher (2008) noted that the type is in Heinrich Friese collections, but we were unable to find it in available museums, including the NHMUK and TMU.

####### Material examined

**(pinned specimens).***Topotypes*: NOVAYA ZEMLYA, YUZHNY ISLAND: Malye Karmakuly, 72.3992°N, 52.8671°E, meadow-like association in tundra, 2♀, Spitsyn leg. [RMBH: voucher nos. BMB88 and BMB90]; Malye Karmakuly, 72.3742°N, 52.7806°E, meadow-like association in tundra, 28.vii.2015, 1☿, Spitsyn leg. [RMBH]; Malye Karmakuly, 72.3754°N, 52.7241°E, meadow-like association in tundra, 30.vii.2015, 1♀, Spitsyn leg. [RMBH]; Malye Karmakuly, 72.3905°N, 52.7167°E, meadow-like association in tundra, 9.viii.2015, 1♀, Spitsyn leg. [RMBH]. *Other recent material examined*: NOVAYA ZEMLYA, YUZHNY ISLAND: Bezymyannaya Bay, 72.8169°N, 53.7843°E, tundra with *Astragalusalpinus*, 21.vii.2017, 1♀, Spitsyn leg. [RMBH]; Bezymyannaya Bay, 72.8338°N, 53.3781°E, tundra with *Astragalusalpinus*, 23.vii.2017, 1♀, Spitsyn leg. [RMBH]; Bezymyannaya Bay, 72.8781°N, 53.6303°E, tundra with *Hedysarumarcticum*, 23.vii.2017, 1☿, Spitsyn leg. [RMBH]; Bezymyannaya Bay, 72.8667°N, 53.6335°E, tundra with *Hedysarumarcticum*, 19–21.vii.2017, 1♀, Spitsyn leg. [RMBH]; Bezymyannaya Bay, 72.8335°N, 53.7339°E, meadow-like association with *Artemisiatilesii* and *Salixlanata*, 19–26.vii.2017, 1♀, Spitsyn leg. [RMBH]. *Historical material examined*: NOVAYA ZEMLYA, YUZHNY ISLAND: Matochkin Shar Strait, 11.viii.1925, 1♀, Pokrovskiy leg. [ZMMU]; Malye Karmakuly, 23.vii.1896, 1☿, 7♂, Jacobson leg. [ZISP]; Kostin Shar Strait, Propashchaya Bay, meadow-like habitat on coast, 9.viii.1913, 1♂, Skribov leg. [ZISP]; Matochkin Shar Strait, 13–15.vii.1924, 1♀, Tolmachev leg. [ZISP]; Matochkin Shar Strait, near broadcast station, 18.vii.1924, 1♀, Tolmachev leg. [ZISP]; Matochkin Shar Strait, Poperechniy Cape, 5.viii.1924, 1☿, Tolmachev leg. [ZISP]. NOVAYA ZEMLYA, SEVERNY ISLAND: Bychkov River, 5.viii.1907, 1♂, collector unknown [ZISP]. NOVAYA ZEMLYA: exact locality and date unknown, 5♀, Pittioni det. [NHMUK].

####### Description of the topotypes.

*Queen morphology*: Malar space slightly longer than the distal width. Central part of clypeus with rather sparse puncturing, while puncturing becomes gradually denser laterally and in the lower part of clypeus. Supra-orbital line transecting ocelli. A3 distinctly longer than A4, A4 shorter than A5. Outer surface of the hind tibia distinctly alutaceous, dull. T4 and T5 chagrinated and punctured. *Queen color pattern*: Head and face black, vertex with slight admixture of yellow hairs. Collar, scutellum, T1 and T2 ochreous-yellow. T3 – T6 black. T6 with slight admixture of ferruginous hairs, which is more distinct in the specimen BMB88.

####### Color variations.

Other specimens collected on Novaya Zemlya (Table 1 and Suppl. material 2, Table S3) share variation in an admixture of ferruginous hairs of T4 – T6. It ranges from black coloring of these tergites without ferruginous hairs to quite distinct ferruginous T4 – T6 in a worker (specimen BMB86). The latter type of coloration clearly matches the protologue of Friese (1902: 495): “Segment 4 – 6 rot behaart (ano rufo)”.

####### Phenology.

**Figure 8.**
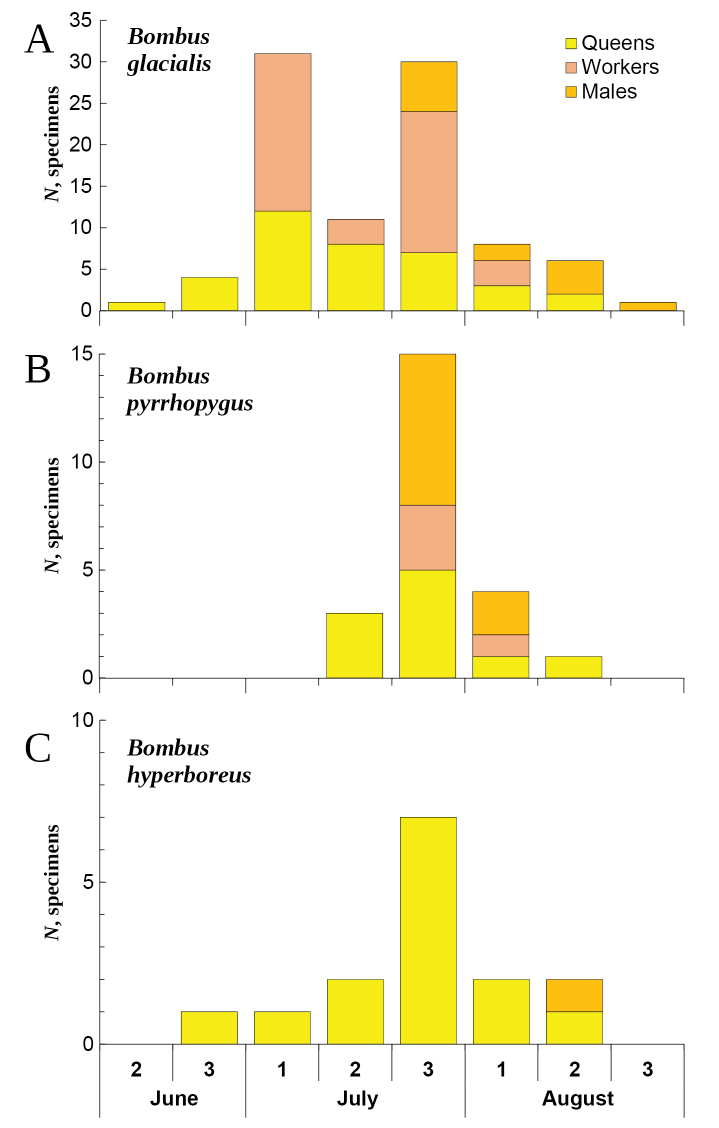
Phenology of bumble bees from Novaya Zemlya by ten-day periods (summary data from the historical and recent samples). (**A**) *Bombusglacialis* (*N* = 92 specimens). (**B**) *B.pyrrhopygus* (*N* = 23 specimens). (**C**) *B.hyperboreus* (*N* = 15 specimens).

This species differs from the other Novaya Zemlya bumble bees by the shortest flight period from mid-July to mid-August, with workers and males emerging in late July (Fig. 8B).

####### Distribution.

Arctic Eurasia from Scandinavia to Chukotka Peninsula (Williams et al. 2015, Williams 2018), including the Yuzhny Island and the southern edge of Severny Island of the Novaya Zemlya Archipelago.

####### Taxonomic comments.

Friese (1902) briefly described this taxon as the subspecies *Bombuskirbyelluspyrrhopygus*. Later, Friese (1905) provided the primary diagnostic features of this subspecies. Currently, *B.pyrrhopygus* was considered a valid species, which is closely related to the Nearctic *B.polaris* Curtis, 1835 (Williams et al. 2015, 2016, Williams 2018). This conclusion is fully supported by our modeling (Fig. 4). Martinet et al. (2018) recently placed this species as a subspecies of *Bombuspolaris* based on the similarity of the major compounds in the male cephalic labial gland secretions (CLGS). However, we disagree with this solution, because the level of genetic distance between these taxa (uncorrected COI *p*-distance = 3.2%) is too high for subspecies-level differences. Here, we consider *Bombuspolaris* and *B.pyrrhopygus* as two separate species.

###### Bombus hyperboreus

Taxon classificationAnimaliaHymenopteraApidae

Schönherr, 1809

98c5b15f-e998-5a4c-a80f-ebe8e1559656

Fig. 7A–E

Bombus hyperboreus Schönherr (1809): 57.

####### Type locality.

Lapponia [Lapland], Sweden

####### Type.

Holotype NHRS-HEVA000004559, Swedish Royal Museum of Natural History (Naturhistoriska riksmuseet), Stockholm, Sweden.

####### Material examined

**(pinned specimens).***Recent material examined*: NOVAYA ZEMLYA, YUZHNY ISLAND: Malye Karmakuly, 72.3992°N, 52.8671°E, meadow-like association in tundra, 27.vii.2015, 1♀, Spitsyn leg. [RMBH]; Bezymyannaya Bay, 72.8528°N, 53.7134°E, tundra with *Astragalusalpinus*, 19–26.vii.2017, 1♀, Spitsyn leg. [RMBH]; Bezymyannaya Bay, 72.8335°N, 53.7339°E, meadow-like association with *Artemisiatilesii* and *Salixlanata*, 19–26.vii.2017, 1♀, Spitsyn leg. [RMBH]. *Historical material examined*: NOVAYA ZEMLYA, YUZHNY ISLAND: exact locality and date unknown, 1♀, Pittioni det. [NHMUK]; Kostin Shar Strait, Propashchaya Bay, 16.viii.1925, 1♀, Pokrovkiy leg. [ZMMU]; Malye Karmakuly, 23.vii.1896, 2♀, Jacobson leg. [ZISP]; Kostin Shar Strait, Propashchaya Bay, 1.viii.1913, 1♀, Skribov leg. [ZISP]; Matochkin Shar Strait, 13–15.vii.1924, 1♀, Tolmachev leg. [ZISP]; Matochkin Shar Strait, slope near Nochuev Stream, 23.vii.1924, 1♀, Tolmachev leg. [ZISP]; Matochkin Shar Strait, near observatory, 29.vi.1925, 1♀, Vakulenko leg. [ZISP]; Matochkin Shar Strait, 6.vii.1925, 12.vii.1925, 2♀, Vakulenko leg. [ZISP]. NOVAYA ZEMLYA, SEVERNY ISLAND: Krestovaya Bay, 10–12.viii.1909, 1♂, Rusanov leg. [ZMMU]; Chekin Bay, 27.vii.1901, 1♀, Timofeev leg. [ZISP]; Novosiltsev Lake, 2.viii.1901, 1♀, Timofeev leg. [ZISP].

####### Phenology.

This species flights from late June to late August, with male appearance in mid-August (Fig. 8C), while its worker caste is lacking throughout the Arctic (Løken 1973; Lhomme and Hines 2018).

####### Distribution.

The nominative subspecies inhabits Arctic Eurasia, including the Yuzhny Island and the southern edge of Severny Island of the Novaya Zemlya Archipelago, while *Bombushyperboreusnatvigi* is known from Arctic North America, and Greenland (Williams et al. 2015, Williams 2018; this study).

**Taxonomic comments on the *Bombushyperboreus* species complex.** Three taxa belong to the *Bombushyperboreus* species complex: *B.hyperboreus* from Arctic Eurasia (including Novaya Zemlya), *B.natvigi* from Arctic North America and Greenland, and *B.kluanensis* from Alaska and Yukon (Williams et al. 2015, 2016). These taxa are phylogenetically close to each other (Fig. 4). While Williams et al. (2015, 2016) considered *Bombusnatvigi* to be a valid species using the COI gene fragment, Martinet et al. (2018) suggested that it is a subspecies of *B.hyperboreus* because of similarity in the major CLGS compounds. We used an expanded data set of COI sequences of *Bombushyperboreus* and *B.natvigi* with two additional intermediate haplotypes from Greenland and USA that filled the molecular gap between these taxa discovered by Williams et al. (2015, 2016). Our mPTP species-delimitation model houses the haplotypes of *Bombushyperboreus*, *B.natvigi*, and *B.kluanensis* within a single MOTU (Fig. 4). Taking into account a shallow genetic divergence between *Bombushyperboreus* and *B.natvigi*, we consider these taxa as two geographic races within the widespread *Bombushyperboreus* that agrees with the CLGS-based concept of *Alpinobombus* developed by Martinet et al. (2018). However, *Bombuskluanensis* shares a rather high level of genetic divergence from *B.hyperboreus* and *B.natvigi* (mean uncorrected COI *p*-distances = 2.1–2.4%), and it must be considered valid species.

###### Subgenus Pyrobombus

Taxon classificationAnimaliaHymenopteraApidae

Dalla Torre, 1880

23241425-2374-5ab9-968e-f2eb426b7d43

####### Type species.

*Apishypnorum* Linnaeus (by monotypy)

###### Bombus glacialis

Taxon classificationAnimaliaHymenopteraApidae

Friese, 1902

fc4a5b96-91ce-5e2d-b2ed-946d7927b87c

Bombus lapponicus subsp. glacialis Friese (1902): 495 [introduced as Sparre-Schneider’s manuscript name]; Friese (1905): 515.
Bombus lapponicus sensu Friese, 1923 non Fabricius, 1793. – Friese (1923): 4.
Bombus lapponicus var. errans Friese, 1923: 4.
Bombus lapponicus var. errans var. aberrans Friese, 1923: 4 [intrasubspecific name (Art. 45.6.1 of ICZN), unavailable (Art. 45.5 of ICZN)].
Pratibombus glacialis Sparre-Schneider, 1902. – Skorikov (1937): 60.
Bombus glacialis Sparre-Schneider, 1902. – Panfilov (1978): 512.
Bombus glacialis Friese, 1902. – Rasmont and Iserbyt (2014); Rasmont et al. (2015): 172; Potapov et al. (2018a): 635.

####### Type locality.

Nowaja Semlja [Novaya Zemlya] (Friese 1902).

####### Type.

Syntype ♀ No. TSZX 7288 labelled “Nova Semlja. v. *glacialis* Sp. Schn.”, Sparre-Schneider’s type collection, Tromsø University Museum, Norway [examined and re-described by us (Potapov et al. 2018a)].

####### Material examined (pinned specimens).

*Recent material examined*: NOVAYA ZEMLYA, YUZHNY ISLAND: Malye Karmakuly, 72.3992°N, 52.8671°E, meadow-like association in tundra, 27.vii.2015, 1♀, 1♂, Spitsyn leg. [RMBH]; Malye Karmakuly, 72.3742°N, 52.7806°E, meadow-like association in tundra, 28.vii.2015, 2♀, 1☿, Spitsyn leg. [RMBH]; Malye Karmakuly, 72.3739°N, 52.7167°E, meadow-like association in tundra, 5.viii.2015, 1♀, Spitsyn leg. [RMBH]; Malye Karmakuly, 72.4229°N, 52.8143°E, meadow-like association in tundra, 6.viii.2015, 1☿, Spitsyn leg. [RMBH]; Bezymyannaya Bay, 72.8338°N, 53.3781°E, tundra with *Astragalusalpinus*, 23.vii.2017, 5☿, Spitsyn leg. [RMBH]; Bezymyannaya Bay, 72.8120°N, 53.8411°E, tundra with *Astragalusalpinus*, 23.vii.2017, 1☿, Spitsyn leg. [RMBH]; Bezymyannaya Bay, 72.8667°N, 53.6335°E, tundra with *Hedysarumarcticum*, 19–21.vii.2017, 1☿, Spitsyn leg. [RMBH]; Bezymyannaya Bay, 72.8528°N, 53.7134°E, tundra with *Astragalusalpinus*, 19–26.vii.2017, 1♀, 6☿, Spitsyn leg. [RMBH]; Bezymyannaya Bay, 72.8335°N, 53.7339°E, meadow-like association with *Artemisiatilesii* and *Salixlanata*, 19–26.vii.2017, 2☿, Spitsyn leg. [RMBH]. *Historical material examined*: NOVAYA ZEMLYA, YUZHNY ISLAND: Matochkin Shar Strait, 12.vii.1925, 1♀, Vakulenko leg. [NHMUK]; Kostin Shar Strait, 19.vii.1895, 1♀, collector unknown [TMU]; Matochkin Shar Strait, near broadcast station, 3.vii.1924, 1♀, Tolmachev leg. [ZMMU]; Matochkin Shar Strait, slope near the mouth of Nochuev Stream, on *Polemoniumboreale*, 31.vii.1925, 1♂, Vakulenko leg. [ZMMU]; Peschanka River, 22.viii.1902, 1♂, collector unknown [ZISP]; Matochkin Shar Strait, near broadcast station, 21.vi.1924, 3.vii.1924, 12.vii.1924, 18.vii.1924, 11.viii.1924, 5♀, Tolmachev leg. [ZISP]; Matochkin Shar Strait, 13–15.vii.1924, 2♀, Tolmachev leg. [ZISP]; Matochkin Shar Strait, slope near Nochuev Stream, 23.vii.1924, 1♀, Tolmachev leg. [ZISP]; Matochkin Shar Strait, Poperechniy Cape, 5.viii.1924, 1♀, 1☿, 2♂, Tolmachev leg. [ZISP]; Matochkin Shar Strait, on *Salixarctica*, 2.vii.1925, 2♀, Tolmachev leg. [ZISP]; Matochkin Shar Strait, on *Saxifragaoppositifolia*, 2.vii.1925, 1♀, Tolmachev leg. [ZISP]; Matochkin Shar Strait, nest of bumble bee, 2.vii.1925, 1♀, 10☿, Tolmachev leg. [ZISP]; Matochkin Shar Strait, Nochuev Stream, on *Astragalusumbellatus*, 18.vii.1925, 2☿, Tolmachev leg. [ZISP]; Matochkin Shar Strait, Nochuev Stream, 1.viii.1925; 1♀, Tolmachev leg. [ZISP]; plateau, 1.viii.1925, 1☿, Tolmachev leg. [ZISP]; Matochkin Shar Strait, coast, 9.vi.1925, 1♀, Vakulenko leg. [ZISP]; Matochkin Shar Strait, slope of Blizhnyaya Mountain, 21.vi.1925, 3♀, Vakulenko leg. [ZISP]; Matochkin Shar Strait, 6.vii.1925, 9.vii.1925, 10.vii.1925, 4♀, Vakulenko leg. [ZISP]; Matochkin Shar Strait, burrow of lemming, 15.vii.1925, 1♀, Vakulenko leg. [ZISP]. NOVAYA ZEMLYA, SEVERNY ISLAND: Verkhnyaya Tyulenya Bay, nest of bumble bee, 9.vii.1901, 9☿, Timofeev leg. [ZISP]; Chekin Bay, 27.vii.1901, 1♀, Timofeev leg. [ZISP]; Krestovaya Bay, 10–12.viii.1909, 1♀, 4♂, Rusanov leg. [ZISP]; Krestovaya Bay, 22.vii.1910, 1♀, 2☿, 4♂, Sosnovskiy leg. [ZISP]; Belushya Bay, 5.vii.1925, 7.vii.1925, 2♀, Vakulenko leg. [ZISP].

**Phenology.** This species has the longest flight period among Novaya Zemlya bumble bees that lasts from early June or mid-June to late August (Fig. 8A). Its workers are appeared in early July, while the flight of males starts in late July.

**Distribution.** Yuzhny Island and the southern edge of Severny Island of the Novaya Zemlya Archipelago, probably also Wrangel Island (Berezin 1990; Chernov 2008; Potapov et al. 2018a). The records from the Kanin Peninsula and Kolguev Island (Poppius 1908; Pittioni 1943) are highly questionable (Potapov et al. 2018a).

**Taxonomic comments.** The results of our previous integrative study indicate that *Bombusglacialis* is a separate bumble bee species that is phylogenetically and morphologically distinct from the other taxa in the *B.lapponicus* complex (Potapov et al. 2018a).

## Discussion

### Bumble bee fauna of Novaya Zemlya with taxonomic remarks on historical checklists

Three species of bumble bees were recorded from Novaya Zemlya based on recent and historical samples: *Bombuspyrrhopygus*, *B.hyperboreus*, and *B.glacialis* (Table 2). These three species were recorded from the Yuzhny Island and the southern edge of Severny Island of the Novaya Zemlya Archipelago up to 74° N (Table 1). This estimation disagrees with previous authors, whose listed two more taxa, i.e., *Bombuskirbyellus* s. lato (= *B.balteatus*) (Friese 1902, 1911a, b, 1908, 1923; Høeg 1924) and *B.lapponicus* (e.g., Friese 1908, 1923; Høeg 1924; Rasmont and Iserbyt 2014).

It is known that old European entomologists often confused *Bombuspyrrhopygus* with *B.balteatus* (= *B.kirbyellus* s. lato) due to the high levels of variability in external coloration patterns (fide Richards 1931). Based on the coloration of the 5^th^ and 6^th^ tergites, Friese (1902, 1908, 1923) recognised three forms of *Bombuskirbyellus*: white tailed, red tailed, and black tailed. The two latter forms were commonly recorded from Novaya Zemlya, while the white-tailed form (typical form of *B.kirbyellus* sensu Friese, 1923) was not found on the archipelago (Friese 1923). Based on the morphological descriptions of Friese (1902, 1908, 1923), his white-tailed form of *Bombuskirbyellus* must be considered *B.balteatus*, while his red-tailed and black-tailed forms represent morphological varieties of *B.pyrrhopygus* (Williams et al. 2015, 2016, Williams 2018). We were also unable to find *Bombusbalteatus* in recent and historical samples from Novaya Zemlya, and this species should not be included to the fauna of the archipelago.

Specimens of *Bombuslapponicus* are also lacking in recent and historical samples from Novaya Zemlya (Tables 1–3 and Suppl. material 2, Table S3), while *B.glacialis* has a quite distinct set of morphological features that allows to distinguish it from *B.lapponicus* (Chernov 2008; Potapov et al. 2018a). Based on this evidence, we can conclude that all historical records of *Bombuslapponicus* and its varieties from Novaya Zemlya (Friese 1908, 1923; Høeg 1924; Rasmont and Iserbyt 2014) actually refer to *B.glacialis*. In this study, we provide an updated synonymy of *Bombusglacialis* that includes one additional subspecific name, i.e., *B.lapponicuserrans*, introduced by Friese (1923) for this biological species.

### Taxonomic comments on the subgenus Alpinobombus

Based on newly obtained results, we suggest that this subgenus includes eight valid species as follows:

(1) *B.alpinus* (Linnaeus, 1758) [supported by the COI (Williams et al. 2015, 2016; this study) and CLGS data (Martinet et al. 2018)]

=*B.alpinushelleri* von Dalla Torre, 1882 [Martinet et al. (2018) placed this taxon as a subspecies of *B.alpinus*. However, its molecular divergence from the Arctic populations is very shallow, and it must be treated as a synonym of *B.alpinus*]

(2)*B.balteatus* Dahlbom, 1832 [supported by the COI (Williams et al. 2015, 2016; this study) and CLGS data (Martinet et al. 2018)]

(3) *B.hyperboreus* Schönherr, 1809 [supported by the COI (Williams et al. 2015, 2016; this study) and CLGS data (Martinet et al. 2018)]

ssp. hyperboreus Schönherr, 1809 [Arctic Eurasia]

ssp. natvigi Richards, 1931 [Arctic North America and Greenland]

(4) *B.kluanensis* Williams & Cannings, 2016 [supported by the high level of the COI divergence (Williams et al. 2016; this study); not supported by the mPTP model (this study)]

(5) *B.kirbiellus* Curtis, 1835 [supported by the COI (Williams et al. 2015, 2016; this study) and CLGS data (Martinet et al. 2018)]

(6) *B.neoboreus* Sladen, 1919 [supported by the COI (Williams et al. 2015, 2016; this study) and CLGS data (Martinet et al. 2018)]

(7) *B.polaris* Curtis, 1835 [supported by the COI (Williams et al. 2015, 2016; this study) and CLGS data (Martinet et al. 2018)]

(8) *B.pyrrhopygus*Friese 1902 [supported by the COI data (Williams et al. 2015, 2016; this study), not supported by the CLGS data (Martinet et al. 2018)]

### Comparison of the bumble bee species richness on Novaya Zemlya with other Arctic areas

Based on our assessment (Table 4), the low number of species on Novaya Zemlya seems to be a rather typical feature for the Arctic insular bumble bee faunas. A much higher species richness of bumble bees in the Icelandic fauna reflects multiple human-mediated dispersal and introduction events (Prŷs-Jones et al. 2016; Potapov et al. 2018b). Several common Eurasian Arctic species are lacking in the fauna of Novaya Zemlya, e.g., *Bombusbalteatus*, *B.lapponicus*, and *B.flavidus*, while these species are known from the nearest Vaygach Island (Potapov et al. 2017). Perhaps, the Kara Strait separating the Vaigach Island from the Yuzhny Island serves as a 50 km wide marine barrier and prevents further expansion of widespread bumble bees to Novaya Zemlya and backward dispersal of *Bombusglacialis* from the archipelago. In contrast, the narrow Matochkin Shar Strait (0.6–3 km wide) between the two main islands of the archipelago does not hamper the dispersal of bumble bees as all the three species were recorded from the Severny Island (Fig. 1).

As for the mainland, sites with the highest number of bumble bee species are situated in river and mountain valleys having species-rich flowering plant associations that allows environment-induced local expansions of boreal bumble bees (e.g., *Bombusdistinguendus*, *B.hortorum*, and *B.consobrinus*) to the Arctic (Shvartsman and Bolotov 2008; Kolosova and Potapov 2011; Potapov et al. 2014). In general, *Bombuslapponicus*, *B.pyrrhopygus*, *B.balteatus*, and the nominative subspecies of *B.hyperboreus* prevail in bumble bee assemblages throughout the Eurasian Arctic, with the exception of Novaya Zemlya. *Bombusglacialis*, in its turn, is the most abundant species on the Yuzhny Island of Novaya Zemlya (Potapov et al. 2018a), and probably on the Wrangel Island (Berezin 1990). *Bombussylvicola*, *B.polaris*, *B.kirbiellus*, and *B.hyperboreusnatvigi* are the most common species in the American Arctic (Proshchalykin and Kupianskaya 2005; Williams et al. 2015; Potapov et al. 2014, 2017, 2018a).

We found that the mean species richness of bumble bees on the Arctic Ocean islands is three times lower than that in the mainland Arctic areas (3.1 *vs.* 8.6 species per local fauna, respectively). Our GLMs revealed that this difference could be explained by specific environmental conditions of insular areas, i.e., the colder climate (lower mean summer temperature) and the prevalence of harsh Arctic tundra landscapes with extremely poor foraging resources. These results support the conclusion of Chernov (2008) that the level of species richness of terrestrial invertebrates (e.g., butterflies and ground beetles) in high latitudes primarily reflects summer temperatures, i.e., the mean temperature of July.

### Historical biogeographic scenarios

*Bombuspyrrhopygus* was described from Novaya Zemlya, and we have sequenced the prospective topotypes of this species from Malye Karmakuly. The topotypes share the same COI haplotype as samples from Norway and Kamchatka, indicating a broad range of this species across the Arctic Eurasia in the Late Pleistocene or Early Holocene. The phylogeographic pattern discovered in *Bombushyperboreus* is similar to that in *B.pyrrhopygus*, with similar haplotypes in Novaya Zemlya and the mainland areas (Fig. 5).

The populations of *Bombusglacialis* from Novaya Zemlya share three COI haplotypes, indicating its long-term persistence on the archipelago that agrees with the hypothesis of Potapov et al. (2018a) that this species may represent a relict Pleistocene lineage adapted to living in the Arctic desert environment. These results indicate that the Yuzhny Island was ice-free during the last glacial maximum and that this remote land could have served as a cryptic glacial refugium for terrestrial and freshwater invertebrates and terrestrial plants (Serebryanny and Malyasova 1998; Mangerud et al. 2008; Coulson et al. 2014; Potapov et al. 2018a; Makhrov et al. 2019). However, several paleogeographic models suggest that Novaya Zemlya was almost completely covered with ice sheet, at least from the mid-Pleistocene (Svendsen et al. 2004; Patton et al. 2016; Ivanova et al. 2016; Hughes et al. 2016).

At first glance, we could assume that *Bombuspyrrhopygus* and *B.hyperboreus* spread across the emerged Eurasian shelf margin in the Late Pleistocene, with subsequent fragmentation of their continuous ranges in the Holocene. *Bombusglacialis* shares another phylogeographic pattern, with at least three unique COI haplotypes in Novaya Zemlya’s population, while this species was not found from the mainland areas (Potapov et al. 2018a). This pattern could be explained by specific environmental preferences of this species, which is clearly linked to the Arctic desert areas (Chernov 2008; Potapov et al. 2008a). This species has the longest flight period (early June or mid-June to late August) among Novaya Zemlya bumble bees that probably reveals its better life cycle adaptation to the hard climate of the archipelago.

### Low abundance of bumble bees on Novaya Zemlya and environmental features

Bumble bees are extremely scarce on Novaya Zemlya, with only a few specimens being collected per sampling effort (Table 1). This seems to be a natural feature of this area, because the mean number of specimens and species per sample does not share significant differences between the historical and recent samples (1895–1925 *vs.* 2015–2017). While the harsh polar climate itself could significantly decrease the abundance of bumble bees (see results of the GLMs above), this phenomenon could have been caused by two additional reasons. First, *Bombusglacialis* and *B.pyrrhopygus* have small colonies producing few workers (Jacobson 1898; Potapov et al. 2018a), while *B.hyperboreus* is a social parasite in the nests of *B.pyrrhopygus* and has no workers (Lhomme and Hines 2018). Second, foraging resources are patchily distributed through mountain tundra landscapes of Novaya Zemlya, and bumble bees are primarily associated with meadow-like and herb tundra communities, occupying rather small and highly fragmented areas (Figs 2–3) (Jacobson 1898; Potapov et al. 2018a; this study). The number of flowering plant species supporting bumble bees on Novaya Zemlya is low, with *Astragalusalpinus*, *A.umbellatus*, *Hedysarumarcticum*, *Oxytropiscampestris*, *Chamaenerionlatifolium*, *Pedicularissudetica*, *Sileneacaulis*, and *Saxifragaoppositifolia* serving as the primary foraging resources (Jacobson 1898; Friese 1908; Høeg 1924; this study). Taking into account the low abundance of bumble bees on Novaya Zemlya, human-mediated loss of natural habitats and climate changes may seriously alter the island populations of these insects in the future.

## Supplementary Material

XML Treatment for Alpinobombus

XML Treatment for Bombus pyrrhopygus

XML Treatment for Bombus hyperboreus

XML Treatment for Pyrobombus

XML Treatment for Bombus glacialis

## Supplementary materials

Supplementary material 1 Tables S1–S2. Lists of the COI gene sequences Data type: molecular sequence data https://binary.pensoft.net/file/320149This dataset is made available under the Open Database License (http://opendatacommons.org/licenses/odbl/1.0/). The Open Database License (ODbL) is a license agreement intended to allow users to freely share, modify, and use this Dataset while maintaining this same freedom for others, provided that the original source and author(s) are credited.Grigory S. Potapov, Alexander V. Kondakov, Boris Yu. Filippov, Mikhail Yu. Gofarov, Yulia S. Kolosova, Vitaly M. Spitsyn, Alena A. Tomilova, Natalia A. Zubrii, and Ivan N. Bolotov

Supplementary material 2 Table S3. Samples of bumble bees from Novaya Zemlya examined in this study Data type: specimen data https://binary.pensoft.net/file/320150This dataset is made available under the Open Database License (http://opendatacommons.org/licenses/odbl/1.0/). The Open Database License (ODbL) is a license agreement intended to allow users to freely share, modify, and use this Dataset while maintaining this same freedom for others, provided that the original source and author(s) are credited.Grigory S. Potapov, Alexander V. Kondakov, Boris Yu. Filippov, Mikhail Yu. Gofarov, Yulia S. Kolosova, Vitaly M. Spitsyn, Alena A. Tomilova, Natalia A. Zubrii, and Ivan N. Bolotov
