# Supplementary material for: Pollinators on the polar edge of the Ecumene: taxonomy, phylogeography, and ecology of bumble bees from Novaya Zemlya
Source: Zookeys. 2019 Jul 24;866:85–115. doi: 10.3897/zookeys.866.35084 (PMC6669216; doi:10.3897/zookeys.866.35084)
Supplement: Supplementary material 1 [file zookeys-866-085-s001.pdf]

Table S1. List of additional *COI* sequences of *Bombus pyrrhopygus*, *B. hyperboreus* ssp. *hyperboreus*, and *B. hyperboreus* ssp. *natvigi* obtained from GenBank and BOLD database for the network analysis

Species	Haplotype code	GenBank/BOLD acc. no.	Locality
Bombus pyrrhopygus	PY1	AF279481	Russia: Kamchatka
Bombus pyrrhopygus	PY2	KF434342	Norway
Bombus pyrrhopygus	PY1	NOAPI563-14	Norway
Bombus pyrrhopygus	PY3	NOAPI641-14	Norway
Bombus pyrrhopygus	PY4	WASPS403-14	Russia: Siberia, Krasnoyarsky Kray
Bombus pyrrhopygus	PY5	WASPS446-14	Norway
Bombus pyrrhopygus	PY6	WASPS456-14	Norway
Bombus pyrrhopygus	PY7	WASPS466-14	Norway
Bombus pyrrhopygus	PY8	WASPS467-14	Norway
Bombus pyrrhopygus	PY9	WASPS471-14	Norway
Bombus hyperboreus hyperboreus	HY3	NOAPI569-14	Norway
Bombus hyperboreus hyperboreus	HY4	AY181107	Norway
Bombus hyperboreus hyperboreus	HY2	WASPS422-14	Russia: Arctic Yakutia
Bombus hyperboreus natvigi	NA1	AY181109	Greenland
Bombus hyperboreus natvigi	NA2	AY181108	Greenland
Bombus hyperboreus natvigi	NA3	KU374724	Greenland
Bombus hyperboreus natvigi	NA3	KU374368	Greenland
Bombus hyperboreus natvigi	NA3	KU373302	Greenland
Bombus hyperboreus natvigi	NA3	KU374134	Greenland
Bombus hyperboreus natvigi	NA3	WASPS704-16	USA: Alaska
Bombus hyperboreus natvigi	NA3	BEECF600-11	Canada: Nunavut
Bombus hyperboreus natvigi	NA4	BBWP646-12	USA
Bombus hyperboreus natvigi	NA5	BEECF714-11	Canada
Bombus hyperboreus natvigi	NA6	BEECF715-11	Canada
Bombus hyperboreus natvigi	NA3	BEECF716-11	Canada
Bombus hyperboreus natvigi	NA3	BEECF730-11	Canada

Table S2. List of additional *COI* haplotypes of the subgenus *Alpinobombus* members obtained from BOLD database for the phylogenetic analysis

**Species**	**BOLD acc. no.**	**Locality**
Bombus alpinus	BBBO067-10	Switzerland
Bombus alpinus	BOMNI066-13	Norway
Bombus alpinus	FBAPB990-09	Italy
Bombus alpinus	WASPS382-14	Sweden
Bombus balteatus	BBWP355-09	Mongolia
Bombus balteatus	GBAH0661-06	Norway
Bombus balteatus	WASPS398-14	Siberia
Bombus balteatus	WASPS399-14	Kamchatka
Bombus balteatus	WASPS423-14	Kamchatka
Bombus hyperboreus hyperboreus	GBAH0671-06	Norway
Bombus hyperboreus hyperboreus	NOAPI569-14	Norway
Bombus hyperboreus natvigi	GBAH0672-06	Greenland
Bombus hyperboreus natvigi	GBAH0673-06	Greenland
Bombus hyperboreus natvigi	BBWP646-12	USA
Bombus hyperboreus natvigi	BEECF714-11	Canada
Bombus hyperboreus natvigi	BEECF715-11	Canada
Bombus hyperboreus natvigi	WASPS1192-18	USA
Bombus hyperboreus natvigi	WASPS1193-18	USA
Bombus kirbiellus	BBWP630-12	USA
Bombus kirbiellus	BCIII086-11	Canada
Bombus kirbiellus	BEECD733-09	Canada
Bombus kirbiellus	BEECD850-10	USA
Bombus kluanensis	SSKUC1493-15	Canada
Bombus kluanensis	WASPS567-15	USA
Bombus kluanensis [Holotype]	BWTWO1194-10	Canada
Bombus neoboreus	BBWP633-12	USA
Bombus neoboreus	BEECF872-12	Canada
Bombus polaris	BBWP608-12	Greenland
Bombus polaris	BEECF598-11	Canada
Bombus polaris	BWTWO1272-10	Canada
Bombus polaris	CNWAH6365-15	Canada
Bombus polaris	GRAFW3101-14	Greenland
Bombus polaris	GRAFW3102-14	Greenland
Bombus polaris	MHBEE049-07	Canada
Bombus polaris	MHBEE056-07	Canada
Bombus polaris	MHBEE073-07	Canada
Bombus polaris	WASPS848-16	USA
Bombus pyrrhopygus	NOAPI641-14	Norway
Bombus pyrrhopygus	WASPS403-14	Siberia
Bombus pyrrhopygus	WASPS446-14	Norway
Bombus pyrrhopygus	WASPS466-14	Norway
Bombus pyrrhopygus	WASPS467-14	Norway
Bombus pyrrhopygus	WASPS471-14	Norway
